# The Negative Effects of Physical Activity Calorie Equivalent Labels on Consumers’ Food Brand Evaluation

**DOI:** 10.3390/ijerph191912676

**Published:** 2022-10-04

**Authors:** Xiaoke Yang, Meiling Hong, Dejin Shi, Qian Chen

**Affiliations:** 1 School of Humanities, Fujian University of Technology, Fuzhou 350002, China; 2 College of Management and Economics, Fujian Agriculture and Forestry University, Fuzhou 350002, China

**Keywords:** PACE labels, brand evaluation, regulatory focus, anticipated enjoyment

## Abstract

(1) Background: To prevent excessive caloric intake, a food labeling strategy is widely adopted by governments. Physical activity calorie equivalent (PACE) labels prove to be effective in reducing calorie intake. However, previous literature has mainly discussed the effect of PACE labels on consumers’ purchase intention for high-calorie foods but has not analyzed whether consumers evaluate food brands negatively after inhibiting the consumers’ purchase intention for high-calorie foods. Therefore, the aims of this study are to explore the negative effects of PACE labels on consumers’ food brand evaluation and the underlying psychological mechanism. (2) Methods: This study manipulated the two calorie-information labeling (standard calorie label and PACE labels) in two studies, involving potato chips and chocolate products. It also adopted a prevention focus and anticipated enjoyment of food consumption variables to detect the moderation effects between consumers’ promotion focus and PACE labels. (3) Results: Results show that compared with calorie labels, PACE labels have a more negative influence on consumers’ food brand evaluation. Furthermore, consumers stimulated by PACE labels develop a stronger prevention focus, thereby reducing their anticipated enjoyment of food brands, and ultimately leading to lower brand evaluations. In addition, when consumers have a promotion focus before choosing food, PACE labels cannot reduce their anticipated enjoyment and food brand evaluation for food brands. (4) Conclusions: While focusing on the negative effect of PACE labels on consumers’ purchase intention for high-calorie foods, it should also be noted that PACE labels have a negative effect on food brand evaluation. Therefore, food enterprises should try their best to activate consumers’ promotion focus through various environmental cues, so as to avoid a double negative effect on consumers’ food purchases and brand evaluations.

## 1. Introduction

The global obesity epidemic has led to an increase in a number of chronic diseases, which is caused by unhealthy eating habits [1,2,3]. This tendency also poses a huge burden on governments’ expenditure on social and healthcare security [4]. The literature has confirmed that one effective way to solve obesity problems is to reduce the caloric intake, in other words, to restrict unhealthy foods consumption [5]. Therefore, how to make consumers shift toward healthy eating habits has received much attention from governments and social organizations [6].

**Figure 1:**
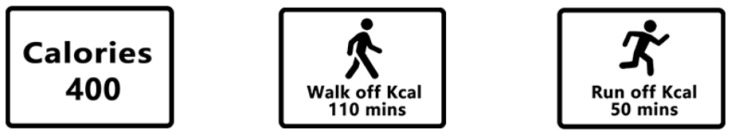
The examples of PACE labels (400 kcal).

Food labeling is a cost-effective strategy to change consumers’ behaviors among nudge inventions, which is adopted by governments to promote healthy eating habits [7,8]. The origin of calorie information was derived from the nutrition facts label, which exhibits the number of calories in certain food [9]. However, some studies showed that the controversial effects of calorie information on lower calorie intakes are caused by consumers not understanding the number of calories [10]. According to Hamlin et al. (2015), consumers only spend six seconds scanning food products [11]. A symbolic label can simplify consumers’ understanding of information and is more effective to change consumer behaviors [12]. The Royal Society for Public Health recently proposed physical activity calorie equivalent (PACE) labels, which display the minutes of different sports needed to burn off the calories of certain foods (Figure 1) [13].

Recently, effects of PACE labels on consumers have been confirmed by the literature. For example, Jin et al. (2020) found that dieters reduce food consumption when exposed to PACE labels [14]. Evidence from multiple systematic reviews by Daley et al. (2021) considered PECA labeling is a promising strategy to help diminish calorie intake [15]. In a study on alcoholic drinks, PACE labeling had a significantly negative influence on participants drinking intentions [16]. Huang et al. (2022) found that unhealthy food with a long time PACE label has a more negative effect on consumers’ purchase intention than a short time PACE label [17]. However, a study by Ellison et al. (2015) pointed out that few studies examine the context in which food consumption takes place; the effect of labels should be put in context, which means involving other factors [18]. As for many packaged unhealthy food products, information labels do not exert influence in consumers purchase intentions alone; brand is a significant factor that people would consider first before making purchase decisions [19,20]. Literature has examined the relationships between information labels and brand evaluation [21,22], and this raises the question whether PACE labels have influence in food brand evaluation, which was rarely discussed before.

Based on the above research conclusions, it can be concluded that PACE labels limit consumers’ calorie intake and purchase intention for unhealthy food to a certain extent. However, after PACE label inhibits consumers’ purchase intention for high-calorie food, how consumers evaluate the food brands needs further study, which directly affects the use of PACE labels and long-term interests of food enterprises. In business practice, only if their commercial interests are satisfied, enterprises can better promote the improvement of consumers’ health and welfare [23].

The research objective is to explore the effects of PACE labels on consumers’ food brand evaluation, and the underlying psychological mechanism further based on the exploratory study. To this end, this study introduces the regulatory focus theory in psychology to analyze whether consumers have different food decision-making tendencies and anticipated enjoyment of food under the stimulation of PACE labels [24,25], thus affecting consumers’ food brand evaluation. Based on the above discussion, the aims of this study are triple: (a) to estimate the effects of different forms of labeling (calorie label and PACE labels) on consumers’ food brand evaluation; (b) to examine the serial mediating mechanism of the effects involving prevention focus and anticipated enjoyment; (c) to determine the moderation effects between consumers’ promotion focus and PACE labels.

The next section of the study shows the hypothesis development of this study; then, the experimental methods are described and results also illustrated in Section 3. We discuss the results and limitations of the study in Section 4, and finally, conclusions are shown in the last section.

## 2. Hypothesis Development 

### 2.1. Regulatory Focus Theory

Based on the regulatory focus theory, the research hypothesis of this paper is deduced. Self-regulation refers to the process through which individuals strive to control or change their thoughts and reactions in order to achieve certain goals [26]. Regulatory focus theory, as a psychological theoretical basis, explains the process of self-regulation. It holds that individuals show a natural tendency to draw on advantages and avert hazards in the process of self-regulation to achieve goals [24,27,28]. It can be divided into promotion focus and prevention focus. The promotion focus is related to the need for advancement, which stimulates more risk-taking behaviors, and pursues positive results to maximize benefits, while the prevention focus is related to the need for security and promotes cautious behaviors, tending to avoid negative outcomes and reduce losses maximally [29,30].

Consumers in the consumption field also follow this self-regulation mode. At the same time, they can adopt different consumption decision-making modes due to various regulatory modes [31]. For example, in the information search stage, those with a promotion focus actively search for information related to gains and successes, forming a larger and more diverse set of considerations, while those with a prevention focus look for information about losses and failures, shaping a smaller and more homogeneous set of considerations [32]. In the process of information processing, those with a promotion focus emphasize speed and usually use exploratory information processing methods, while those with a prevention focus emphasize accuracy and often adopt cautious information processing methods [33]. As illustrated, the self-regulation mode may affect the tendency of consumers to make final decisions. Therefore, it is important to pay attention to the self-regulation mode adopted by consumers in the decision-making process.

In addition, the regulatory focus can be also divided into chronic regulatory focus and situational regulatory focus [34]. The chronic regulatory focus reflects the stable personal trait in self-regulation, while the situational regulatory focus is a temporary tendency induced by situational factors (cue information from the individual’s environment or task frame, etc.) [35,36], which can be stimulated by directing subjects to focus on opportunities or risks [37]. Therefore, this study believes that calorie and PACE labels, as cues about caloric risk, cause to change in consumers’ situational regulatory focus, so that consumers with a promotion or prevention focus change their subsequent food decision-making behavior and food brand evaluation.

### 2.2. The Effects of PACE Labels on Food Brand Evaluation

As mentioned before, previous studies have focused on the negative effects of PACE labels on high-calorie foods [14]. At the same time, many studies have concluded that PACE labels are more effective at reducing consumers’ purchase intention for unhealthy food [21]. However, whether psychological changes in consumers, with their subsequent effect of food decision-making, are produced in the process of PACE labels reducing consumers’ calorie intake and purchase intention for unhealthy food still needs further study. This study explores the effects of PACE labels on consumers in addition to food decision-making, which can be divided into two levels: The first level is that PACE labels may lead consumers to produce negative emotions. For example, it was found that PACE labels reflect a longer time to consume the calories, which can give consumers greater time pressure and calorie risk assessment [38], and the stronger perception of potential calorie risk leads to a higher level of negative emotion predicted for unhealthy food [39], leading to a stronger negative effect on the consumers’ purchase intention for unhealthy food [40,41]. Therefore, PACE labels not only reduce consumers’ high-calorie food intake but also increase consumers’ perception of calorie risk in food and produce psychological changes in consumers, besides food decision-making, related to time pressure and negative emotions.

The second level is that PACE labels may cause consumers to feel anticipatory guilty about their food experience. When people make purchase decisions, they are stimulated by positive or negative emotions [42,43]. For example, consumers experience positive emotions when consuming fried foods [44]. However, certain people (e.g., dieters or sick people) may experience anticipatory guilt when purchasing foods that are high in fat, sugar or calories, because of a conflict with their health or weight goals. People feel guilty about eating a piece of cream cake if they need to lose weight [45]. In general, people are prone to feel guilty when they perceive a relationship between health risks and food consumption [46]. Meanwhile, information can trigger different emotions in the consumption scenarios, including symbols or slogans [47]. Therefore, PACE labels act as a reminder of this health risk, making consumers feel anticipatory guilt about their food experience.

In previous studies, it was found that contextual or task-framing cue information induces a situational regulatory focus in consumers [35,36]. The perception of calorie risk, negative emotions, or anticipatory guilt caused by PACE labels all belong to the potential threat of risk type [37]. Thus, combined with previous studies on PACE labels, it is believed that PACE labels are likely to cause consumers to develop prevention focus, rather than promotion focus. In addition, consumers with prevention focus make more conservative and negative evaluations on the product and its surroundings [48], thereby not choosing the product. However, those with a promotion focus have an obvious conservative tendency, and prefer to adopt a vigilance strategy, which reduces the losses caused by actions, and thus weakens the advantages of the product [49]. Furthermore, according to cognitive dissonance theory [50], consumers can avoid purchasing the item by lowering their evaluation of the target subject.

To sum up, this study holds that PACE labels stimulate consumers’ prevention focus in food decision-making, thereby making it more conservative and cautious. To avoid the risk posed by the product, the choice of high-calorie food is reduced, but in order to justify the choice, the brand evaluation of the food is downgraded so as to achieve cognitive-behavioral alignment. Therefore, compared with traditional food labels, such as calorie labels, PACE labels cannot only reduce consumers’ purchase intention for high-calorie foods but also bring about a stronger negative effect on consumers’ food brand evaluations. Based on the above discussion, the following assumptions are made:

**Hypothesis** **1** **(H1).** *Compared with calorie labels, PACE labels have a stronger negative effect on consumers’ food brand evaluation.*

### 2.3. Serial Mediation Model Involving Prevention Focus and Anticipated Enjoyment

In order to deeply explore the adverse effect caused by PACE labels, we firstly explored the psychological mechanism behind the adverse effect on consumers. In the above discussion, through literature analysis, it is inferred that PACE labels stimulate consumers’ prevention focus, thereby reducing consumers’ food brand evaluation. However, this study believes that there is an important consumer psychological link between the prevention focus and food brand evaluation, that is, consumers’ anticipated enjoyment of food experience.

Anticipated enjoyment motivation is one of the significant factors in consumers purchasing food [25]. In other words, consumers buy food, especially high-calorie food, for anticipated enjoyment, such as pleasure. Consumers’ anticipated enjoyment of food comes largely from the flavor of the food itself, so that a better taste develops higher anticipated enjoyment from food [51]. According to the principle of willingness-behavior consistency, when consumers want to buy high-calorie food, they think that high-calorie food is delicious; however, when consumers are not willing to buy high-calorie food, they lower their taste evaluation on the food [52].

The core of consumers’ self-regulation lies in drawing on advantages and averting hazards [27,28]. Consumers with a prevention focus tend to avoid negative outcomes and reduce losses maximally [30], which can amplify the risks of the product and negative information about it [32,48] and weaken its advantages [49]. In food consumption, consumers think that one of the greatest advantages of food is the anticipated enjoyment of food experience, which is likely to be reduced due to the self-regulation of the prevention focus. Furthermore, related studies show that a lower evaluation of taste may lead to a lower anticipated enjoyment and then cause customers’ purchase intention for food to reduce [53]. Meanwhile, this study believes that as consumers’ anticipated enjoyment of food experience decreases, both the purchase intention for food and the food brand evaluation decline, so as to avoid purchasing the food and achieve the consistency of cognition and behavior [50]. For example, it was found that when food labels make consumers perceive that food has a lower anticipated enjoyment, consumers’ overall food brand evaluation decreases further [54]. Hence, a serial mediation model is built, and the following hypothesis is proposed in this study:

**Hypothesis** **2** **(H2).** *Compared with calorie labels, PACE labels elicit a stronger consumers’ prevention focus, which leads to a decrease in consumers’ anticipated enjoyment of the food, further lowering brand evaluations of the food.*

### 2.4. Moderator Boundary of Promotion Focus

Promotion focus is an important conceptual carrier of self-regulation theory. Generally, scholars believe that individuals with a promotion focus tend to adopt approaching strategies in the process of achieving their goals, that is, they expect to obtain more opportunities to achieve their goals through active exploration [55,56,57]. At the same time, the promotion focus regulates the individual’s cognition of food, so as to act more in pursuit of the inner “ideal self” [58]. Based on the regulatory focus theory, different contents of information can motivate users to have various regulatory focuses [59]. For instance, consumers with a promotion focus pay much attention to the advantages of the product, thereby improving their evaluation of it [60]. In addition, they can adopt heuristic strategies. When reaching the target decision, they mainly use favorable information to make judgments and ignore negative information [61].

**Figure 2:**
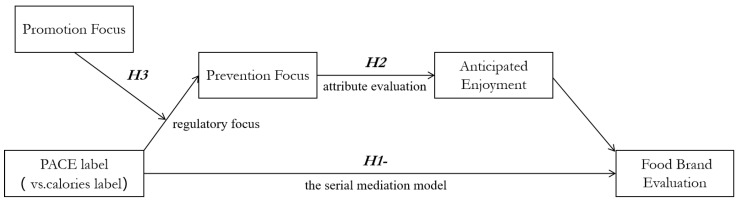
The main framework of the study.

If consumers adopt a prevention focus because of PACE food labels, it reduces their food brand evaluation. Whether this negative effect can be eliminated by stimulating consumers’ promotion focus will be discussed further in this study. Since consumers are affected by cues from the environment or task frame to produce different regulatory focuses [36], this study holds that the reverse self-regulatory manipulation, that is, stimulating consumers’ promotion focus, can eliminate the negative effect on the brand caused by the prevention focus. Therefore, based on the above discussion, this study proposes the following hypothesis:

**Hypothesis** **3** **(H3).** *Consumers’ promotion focus will moderate the negative effect of PACE labels on food brand evaluation. The main framework of the present study was shown in Figure 2.*

## 3. Materials and Methods

In two scenario-based experiments, a variety of stimulus materials were employed to test three hypotheses (Table 1). The best strategy for demonstrating the causal connection between variables is through experimentation [62,63]. Situational simulation experiments were mostly employed in order to remove interference brought on by the real-world environment [64]. By altering the experiment’s test items, potential confounding variables and alternate hypotheses were ruled out, enhancing both the external and internal validity of research findings [65]. Finally, we strictly adhered to the criteria of consumer experiments in all of our data processing techniques and procedures [66,67].

### 3.1. Study 1: Primary Examination of Main and Mediating Effects

This study employs an intergroup design with PACE labels and calories labels for comparison. We anticipated that PACE labels, as opposed to calories labels, would have a higher negative impact on brand appraisal for unhealthy foods. In addition, we also verified that consumers’ demographic had no major influence on the effects of PACE labels on brand evaluation of foods which are unhealthy. Finally, at the conclusion of the experiment, we asked the participants to recall the label content of the stimulus and fill in the blanks, excluding the careless people, to evaluate whether they actually have a reading about the experimental stimulus and know the meaning of the label.

#### 3.1.1. Methodology

Top institutions all over the world use the Credamo online platform to provide services because it is a professional data survey. In addition, it is useful for collecting data information from numerous reputable journal papers [68]. In this study, 330 high-reliability responders (Credamo users with credit scores above 90) were chosen at random from the database and were assigned to either the PACE label or the caloric label. Random sampling was used to maintain as much uniformity as possible between the properties of the goods in the various trial groups, reducing the impact of item demographics on experimental results.

If a “Z” law exists, the majority of the items get either the extreme values of 1 or 7 or their labels are not remembered. A total of 26 samples from the study were eliminated, leaving 304 valid questionnaires (Table 2 lists the demographics of the individual samples), including 148 respondents for the PACE label group and 156 respondents for the caloric label group. Each group’s effective sample size exceeds 50 and achieves its standards, as per the rules of consumer behavior experiment [69].

#### 3.1.2. Procedure and Materials

**Figure 3:**
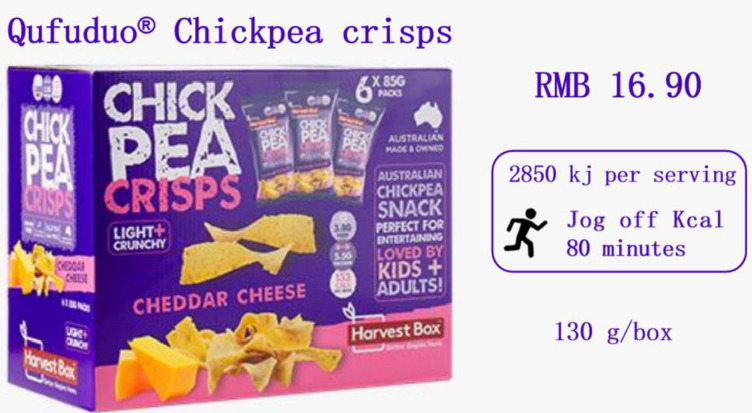

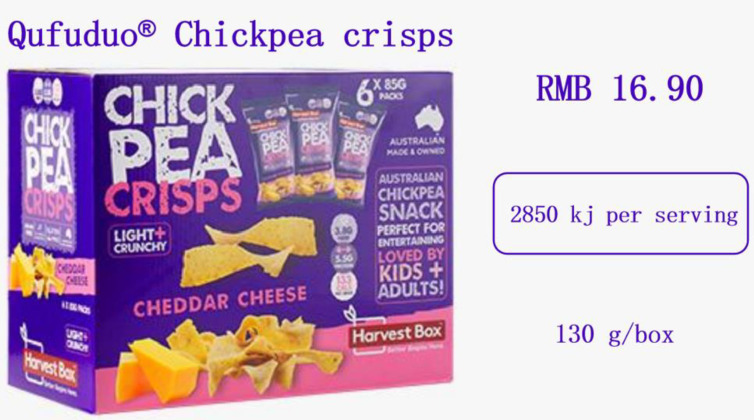
Stimuli of study 1.

One of the two groups received a random assignment of participants. Prior to evaluating and scoring chickpea crisps, participants read the fundamental information. The experiment’s fictional product, Qufuduo (130 g/bag, 16.9 yuan), is due to be released by a food firm. This is the product’s background information. Regarding the two groups, the PACE label and calories label were affixed to the product introduction of chickpea crisps; otherwise, the two labels were identical. The following are the two labels’ precise specifications (Figure 3): The words “calorie label” and “PACE label” stand for “2850 kj per serving” and “jogging for 80 min is required to burn off the calories,” respectively. The contrasting paradigms PACE and calorie labels refer to previous mature studies [17].

Second, after reading the fundamental facts about chickpea crisps, participants had to respond to a series of questions to indicate if they intended to buy the product and how they felt about the brand. A 7-point scale was used to score the participants’ purchase intentions, with 1 being a firm no-buy response and 7 representing a definite yes (Huang et al. 2021) [54]. These measurement items of brand evaluation were adapted from Brakus et al. (2009) [70] and minor modifications made in accordance with the research scenario. Thirdly, participants assessed the prevention focus after being stimulated by the food label and its product introduction, and Wan’s (2009) measurement item was modified [49]. Participants also assessed how much they would likely appreciate this chickpea. The Gomez and Torelli scale was used to create this measurement item (2015) [71]. Finally, the demographic characteristics of the participants were measured.

#### 3.1.3. Results

**Figure 4:**
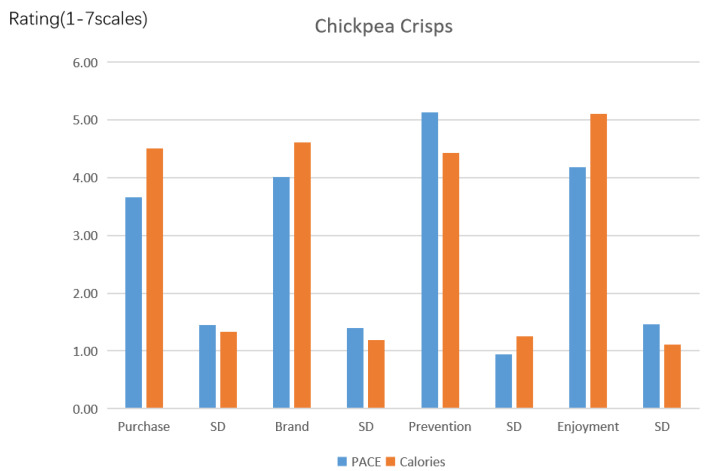
The effect of labels on purchase intention, food brand evaluation, prevention focus and anticipated enjoyment.

Purchase intention. Table 3 and Figure 4 show the experimental outcomes of this investigation. The impacts of various labels on customers’ purchase intentions for junky food were shown by a one-way analysis of variance (ANOVA) (F(1, 302) = 27.988, *p* < 0.001, partial η^2^ = 0.085). F indicates the findings of the F-test about the two groups’ consumers’ intentions to purchase unhealthy food. In terms of the desire of consumers to buy junky food, it is demonstrated that the two groups differ significantly from one another without having the same normal distribution. The experiment’s effect size is partial η^2^. The independent variable affects the dependent variable negligibly if the partial η^2^ value is greater than 0.01. If the partial η^2^ is greater than 0.06, the effect magnitude is considered to be moderate. The effect size is large if the partial η^2^ is greater than 0.14. To determine whether the experiment’s sample size was adequate, we also performed a power analysis on the data. 1-β was written as 1.000 in this test, suggesting that the size of the experiment sample is adequate.

The *t*-test was adopted to further assess the mean values of the calories and PACE label groups. The findings demonstrate that consumers are less likely to make a purchase when the PACE label is shown on the front of a package of chickpea crisps rather than the calories label (MPACE = 3.66, SD = 1.455 vs. MCalories = 4.51, SD = 1.327, *p* < 0.001). The *t*-test shows that the average value of the PACE label is substantially lower than the group using the calories label. This conclusion corresponds to the finding in previous literature that compared it with calorie labels. Consumers are more strongly discouraged from eating calories by PACE labeling [14,15].

Food brand evaluation. The data analysis approach is identical to the one described previously. The impacts of various labels on the evaluation of food brands were shown via a one-way ANOVA (α = 0.920) (*F*(1, 302) = 27.327, *p* < 0.001, *partial η*^2^ = 0.051, and 1-β *=* 0.980). Consumers’ opinions of food brands were found to be less favorable when the PACE label for chickpea crisps was utilized (vs. calories label) (M_PACE_ = 4.01, SD = 1.400 vs. M_Calories_ = 4.61, SD = 1.189, *p* < 0.001). As a result, the validity of Hypothesis 1 was confirmed.

Prevention focus. The impacts of different labels on preventative focus were revealed by a one-way ANOVA (α = 0.837) (*F*(1, 302) = 29.787, *p* < 0.001, *partial η*^2^ = 0.090, and 1-β *=* 1.000). It has been discovered that when the PACE label of chickpea crisps is applied, consumers place a greater emphasis on prevention (vs. calories label) (M_PACE_ = 5.13, SD = 0.947 vs. M_Calories_ = 4.43, SD = 1.250, *p* < 0.001). 

Anticipated enjoyment. Distinct labels had different effects on predicted enjoyment, according to a one-way ANOVA (α = 0.922) (*F*(1, 302) = 38.650, *p* < 0.001, *partial η*^2^ = 0.113, and 1-β *=* 1.000). Customers’ expectations of enjoyment are found to be lower when chickpea crisps bearing the PACE label are consumed (vs. calories label) (M_PACE_ = 4.18, SD = 1.458 vs. M_Calories_ = 5.10, SD = 1.115, *p* < 0.001).

**Figure 5:**
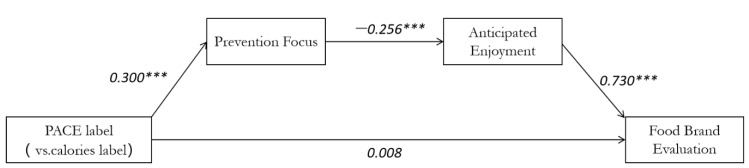
Study1: Mediating effect of prevention focus and anticipated enjoyment between PACE label and brand evaluation. Note: Numbers indicate beta values, and numbers indicate the main effect after adding the mediator variable. *** *p* < 0.001.

Mediation Analysis. Labels were denoted by the codes 1 for PACE and 0 for calories. The impact of the PACE label on the brand rating was then mediated by measures of preventative focus and expected enjoyment. Additionally, a serial mediation model investigated the particular hypothesized pathway. The behavioral paradigm was closely followed in the investigation of the model’s mediating effect [72]. A bootstrap analysis with 5000 samples using Model 6 found that the full serial mediation model utilizing both preventative emphasis and expected enjoyment was important for these (Model 6 features a continuous link between two mediation variables, one independent variable and one dependent variable) fried chickpeas (indirect effect = 0.1015, SE = 0.0420, and 95% CI = −0.1935 to −0.0301) [73]. The absence of 0 in the interval range of the CI value indicates that the mediating impact is considerable [66]. Additional analyses, however, revealed that the “reverse” model (PACE label anticipated enjoyment prevention focus brand evaluation) had no appreciable impact on the chickpea crisps (indirect effect = 0.0094, SE = 0.081, 95% CI = −0.0036 to 0.0283). When the two mediations are performed in reverse order, the mediating impact is insignificant because the interval range of the CI value spans 0. More specific analysis revealed that none of the routes were insignificant (all *p* < 0.001). It indicates the expectation was reached following the direction. We also confirmed the mediation effect once again using the regression coefficient (Figure 5). This shows that the mediation model has a full mediation effect. Additionally, the outcomes corroborate Hypothesis 2.

Demographic characteristics. The trial must rule out the influence of demographic factors on the PACE label. Gender’s primary influence on brand appraisal is shown to be insignificant (F(1, 302) = 3.226, *p* > 0.05), and the PACE label has no interaction impact (*p* > 0.05), suggesting that the impact of the PACE label is largely consistent across gender groups. Three education levels are classified: undergraduate, postgraduate and above, and college/high school and below. The findings indicate that neither the main effect of education on brand rating (F(1, 301) = 0.172, *p* > 0.5) nor any interaction with the PACE label (*p* > 0.1) are present. The income was broken down into four categories: under 2000, 2001 to 4000, 4001 to 6000, and 6000 and above. The findings indicate that there is a strong main influence of income on brand appraisal (*F*(3, 300) = 3.545, *p* = 0.015). Among them, the high-income group (4001–6000, 6000 and above) has stronger brand evaluation than the low-income group (2000, 2001–4000). However, there is no statistically significant interaction between the PACE label and income (*p* > 0.1). As a result, even while different socioeconomic groups evaluate unhealthy food brands differently, the impact of the LPACE label is constant across income levels. Less than 20, 21–30, 31–40, and 41 and over years of age were separated into four categories, and the brand evaluation was regressed. The findings demonstrate that age has no significant impact on brand appraisal (F(3, 300) = 1.414, *p* > 0.1) and that there is no interaction between age and the PACE label (*p* > 0.5). Three BMI ranges were used: 18.4, 18.5–23.9, and 24.0. The findings indicate that neither the BMI nor the PACE label interact to affect how well a brand is perceived (F(2, 301) = 0.737, *p* > 0.1). Because of this, the moderating or interacting impact of participants’ demographic traits on the assessment of the brand with the PACE label was disregarded, and this shows that the negative effects of the PACE label on food brand evaluations can be stable across different groups.

### 3.2. Study 2: Examination of Moderation Effects

Based on a chocolate eating scenario, Study 2 verified the main effect’s boundary requirement (Hypothesis 3). When consumers are the main focus of the promotion, the detrimental impacts of PACE labels on the appraisal of food companies are projected to vanish. The crucial boundary effect of customers’ promotion focus was confirmed using the floodlight method. Additionally, this study believes that the negative influence of PACE labels on the food brand evaluation of consumers with a promotion focus can be eliminated, while the negative effect of the labels on the desire of consumer to buy high-calorie food cannot be significantly detected. Consequently, the two intergroup labels (PACE vs. calories) and two intergroup focus groups (promotion vs. control) design was used, in a two-by-two mixed way. The putative food-related mediation mechanism was also removed at the same time.

#### 3.2.1. Methodology

A total of 400 high-reliability participants in the study were randomly assigned to one of the four experimental groups, which are given below, and were drawn from the Credamo online platform: Promotion-PACE (89 respondents in this group), Promotion-Calories (89 respondents), Control-PACE (90 respondents) and Control-Calories (92 respondents). Finally, a total of 360 valid questionnaires that were collected after 40 samples were excluded for being outliers or failing to recall the labels.

#### 3.2.2. Procedure and Materials

**Figure 6:**
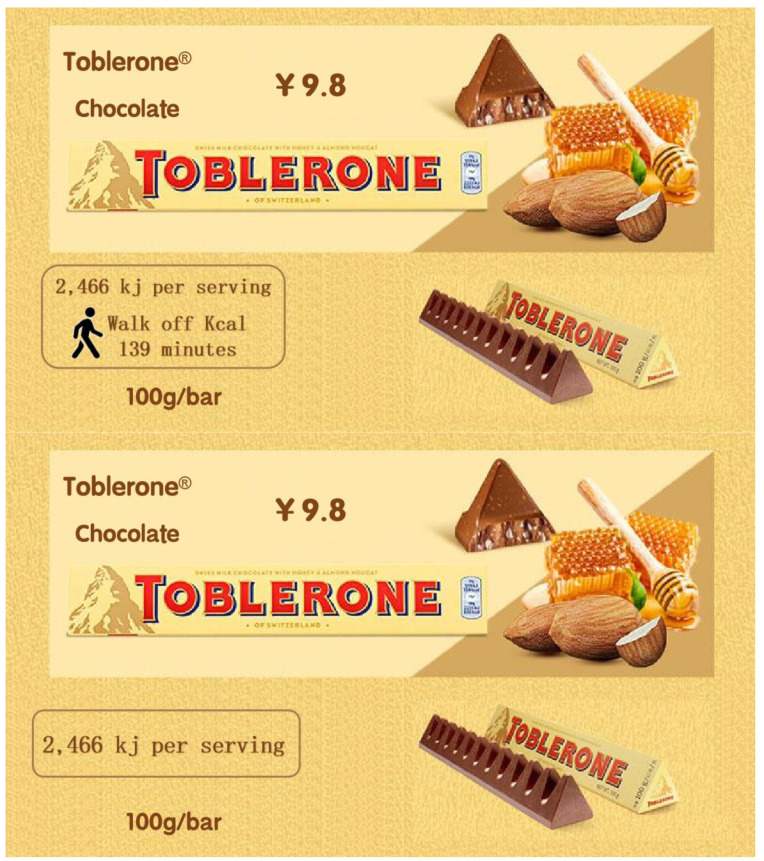
Stimuli of study 2.

Four groups were chosen at random for the participants “Promotion-PACE, Promotion-Calories, Control-PACE and Control-Calories”. They had to be knowledgeable about Toblerone in order to estimate and grade it properly. A food manufacturer is preparing to create a new Toblerone (100 g/bar, 9.8 yuan) as the context for this experiment. The PACE and calories labels were used as the ones for “Toblerone”, where the calories label reads “2466 kj per serving, and it takes 139 min of walking to burn off the calories” (Figure 6).

Writing tasks were employed in this study, and the promotion focus was manipulated. To manipulate the regulatory emphasis, participants were instructed to speak or write [74]. In the promotion focus group, participants had to respond to the following inquiries: “What has been the most important task in your work or life recently (no less than 4 words)?”, and “Which method do you use to get the best possible results in this task? Please briefly write this method (no less than 8 words)”. In the control group, in order to keep their cognitive resource consumption consistent, the participants in the two groups were required to answer the question “What has been the most impressive thing in your work or life recently? Please list two things (no less than 12 words)”.

A series of questions were posed to the participants after reading the basic information about Toblerone in order to convey their purchase intention and brand evaluation of this product. Participants evaluated their regulatory focus after being stimulated by the food label and its product introduction [49]. Participants assessed how much fun they thought they would have with this Toblerone. Finally, the demographic characteristics of the participants were measured.

#### 3.2.3. Results

Manipulation check. As shown in the Table 4. A comparison was made between the promotion focus group and the control group following manipulation, to find out whether the degree of promotion focus (α = 0.829) is different. The results show that the promotion focus group has a substantially higher level of focus than the control group does (M_promotion_ = 5.98, SD = 0.620 vs. M_control_ = 5.18, SD = 1.196, *p* < 0.001, *partial η*^2^ = 0.151, and 1-β *=* 1.000). This conclusion shows that regulatory attention has been successfully manipulated.

**Figure 7:**
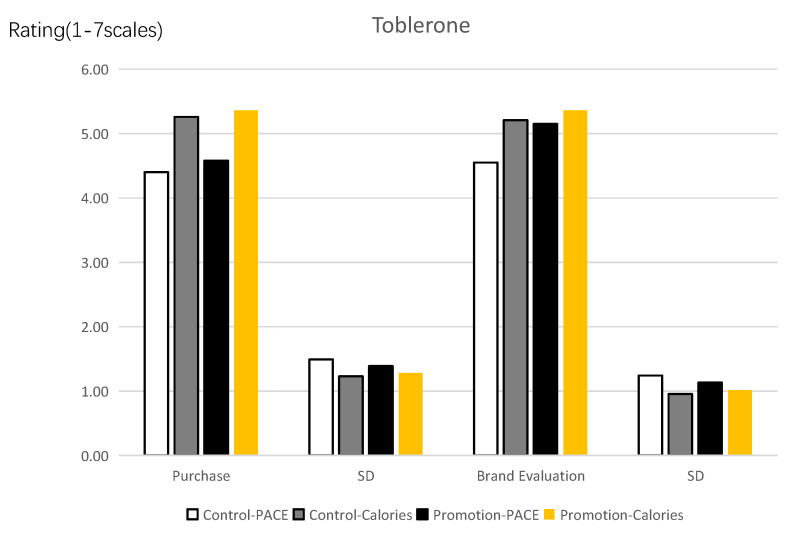
Effects of labels on the purchase intention and food brand evaluation.

Purchase intention. Figure 7 clearly explains the results of this experiment. ANOVA analysis of variance post hoc test was carried out on the desire of participants to buy junky food. The post hoc test shows that in the control group, the desire of participants to buy junky food with the PACE label is significantly lower than with the calories label (M_PACE_ = 4.40, SD = 1.490 vs. M_calories_ = 5.26, SD = 1.230, F(1, 180) = 18.098, *p* < 0.001, *partial η*^2^ = 0.091, and 1-β *=* 0.988). In the promotion focus group, the desire of participants to buy junky food with the PACE label is also significantly lower than with the calories label (M_PACE_ = 4.58, SD = 1.388 vs. M_calories_ = 5.36, SD = 1.281, F(1, 176) = 14.987, *p* < 0.001, *partial η*^2^ = 0.078, and 1-β *=* 0.971). The findings show that even when consumers are in a state of high attention, the detrimental impact of the PACE label on their propensity to buy unhealthy food remains.

Food brand evaluation. ANOVA analysis of variance post hoc test was carried out on the participants’ brand evaluation (α = 0.853) for unhealthy food. The post hoc test shows that in the control group, the participants’ brand evaluation for unhealthy food with the PACE label is significantly lower than with the calories label (M_PACE_ = 4.55, SD = 1.241 vs. M_calories_ = 5.21, SD = 0.954, F(1, 180) = 16.313, *p* < 0.001, *partial η*^2^ = 0.083, and 1-β *=* 0.980). However, when the participants have a promotion focus, there is no significant difference between participants’ brand evaluation in the PACE label group and in the calories label group (M_PACE_ = 5.15, SD = 1.131 vs. M_calories_ = 5.36, SD = 1.017, F(1, 176) = 1.692, *p* > 0.1). It is shown that the promotion focus plays a role of moderate effect in the negative effect of the PACE label on brand evaluation for unhealthy food. When consumers have a promotion focus, the negative effect of the PACE label on brand evaluation for unhealthy food is not significant.

Prevention focus. ANOVA analysis of variance post hoc test was carried out on the participants’ prevention focus (α = 0.826). The post hoc test shows that in the control group, the participants’ prevention focus with the PACE label is significantly higher than with the calories label (M_PACE_ = 5.14, SD = 1.108 vs. M_calories_ = 4.11, SD = 1.601, F(1, 180) = 25.271, *p* < 0.001, *partial η*^2^ = 0.123, and 1-β *=* 0.999). However, when the participants have a promotion focus, there is no significant difference in participants’ prevention focus in the PACE label group and in the calories label group (M_PACE_ = 4.12, SD = 1.637 vs. M_calories_ = 4.10, SD = 1.580, F(1, 176) = 0.009, *p* > 0.5).

Anticipated enjoyment. ANOVA analysis of variance post hoc test was carried out on the participants’ anticipated enjoyment (α = 0.898). The post hoc test shows that in the control group, the participants’ anticipated enjoyment with the PACE label is significantly lower than with the calories label (M_PACE_ = 4.84, SD = 1.526 vs. M_calories_ = 5.63, SD = 1.063, F(1, 180) = 16.610, *p* < 0.001, *partial η*^2^ = 0.084, and 1 − *β* *=* 0.982). However, when the participants have a promotion focus, there is no significant difference in participants’ anticipated enjoyment in the PACE label group and in the calories label group (M_PACE_ = 5.48, SD = 1.103 vs. M_calories_ = 5.75, SD = 0.939, F(1, 176) = 3.256, *p* > 0.05). 

Mediation Analysis. The hypothesized relationship was examined using a moderated mediation analysis. To be more precise, while the PACE label was chosen as the predictor variable, the promotion focus and anticipated enjoyment were chosen as the moderator and mediator, respectively. The complete model was significant on the basis of the bootstrap analysis using Model 7 and 5000 samples (moderated mediation) (R^2^ = 0.1929, and *p* = 0.0001). Additionally, the model is significant, as indicated by the *p* value, and the R^2^ indicates that the model has a reasonable degree of fit. More detailed investigation revealed that the influence of the PACE label on brand appraisal for the promotion focus group was mediated by the expected enjoyment (indirect effect = 0.2889, SE = 0.1522, 95% CI = 0.0607 to 0.6494), rather than the control group (indirect effect = −0.0163, SE = 0.1071, 95% CI = −0.1994 to 0.2247). As a result, Hypothesis 3 is supported.

The preventative focus and anticipated enjoyment were the subjects of a serial mediation study because the model above did not permit a combined serial and moderation analysis. The whole serial mediation model was significant for the control group (indirect effect = 0.1205, SE = 0.0861, 95% CI = 0.0026 to 0.3363), and the “reverse” model was not significant (indirect effect = 0.0085, SE = 0.0211, 95% CI = −0.0347 to 0.0528). As a result, the logic of the mediation order was confirmed and was in line with a serial mediation analysis of Study 1. However, this model was insignificant in the promotion focus group scenario (indirect effect = 0.0001, SE = 0.0121, 95% CI = −0.0205 to 0.0295).

Promotion focus. Further research was carried out on the moderating impact of consumers’ promotion focus. Because the moderator was a cardinal variable, the Johnson–Neyman floodlight analysis technique [75] was used to evaluate the detrimental impact across the board of consumers’ promotion emphasis. By using PROCESS Model 1 [76], the floodlight analysis was completed. (Under various moderating variables, Model 1 reflects the test of causality between independent factors and dependent variables.) The independent, moderator, and dependent variables in Model 1 are contained in PROCESS, a device for moderating based on route analysis. With a 95% CI, the PACE label’s detrimental effects were significant for promotion values greater than 5.0791 (52.71% of the participants) and less than 1.6123 but not for values between 5.0791 and 1.6123. Those with a value lower than 1.6123 made up just 0.7752% of the population, however. Therefore, at a promotion focus value below 5.0791 (47.29%), the effect was inoperative (*p* > 0.05). Hypothesis 3 is supported by the overall interaction effect’s significance (*p* = 0.0067). In summary, consumers’ brand evaluation of the PACE label (vs. calories) and other brands had no appreciable differences in the setting of consumer promotion focus (<5.0791). If the number is greater than 5.0791 (52.71%), the consumer may have a promotion-focused mindset. The floodlight method gave the following related research a promotion focus threshold.

Control variable. Consumer’ involvement in food choices refers to the extent to which consumers invest energy, time, and cognitive resources in the process of food selection. In order to reduce the possibility of different consumers’ involvement in food choices due to different food labels, thus leading to different brand evaluations, the mediating effect of consumer’ involvement in food choices other than anticipated enjoyment was also excluded in this study. Consumer’ involvement in food choices had no significant difference in the four groups (M_promotion-PACE_ = 5.02, SD = 1.314 vs. M_control-PACE_ = 5.09, SD = 0.956 vs. M_promotion-calories_ = 5.08, SD = 1.272, M_control-calories_ = 5.18, SD = 1.157, F(3, 356) = 0.295, *p* > 0.5), hence excluding the possibility that customer input in food selection could have an impact on the detrimental impacts of the PACE label.

## 4. General Discussion

This study proved three hypotheses through two experiments. Results showed that compared with the calorie label, the PACE label has more negative effect on consumers’ brand evaluation for unhealthy food. Meanwhile, the prevention focus and anticipated enjoyment of food consumption variables were in the serial mediation model. In addition, consumers’ promotion focus is used as the moderating boundary of this negative effect. 

In previous studies, many scholars have mainly explored the inhibitory effect of PACE labels on consumers’ high-calorie intake [14,15]. It is found that compared with traditional calorie labels, PACE labels have a stronger negative effect on consumers’ calorie intake [16]. In order to further analyze the category of this negative effect, other scholars have expanded from food [19] and PACE label types [21]. It is shown that the PACE label has an inhibitory effect on consumers’ purchase intention for unhealthy food but a promotion effect for healthy food [21]. Meanwhile, the manifestation of the PACE label has different degrees of influence on the negative effect. These conclusions provide a research basis for the negative effect of the PACE label on consumers’ purchase intention for unhealthy food. However, does the PACE label have an associated negative effect when it inhibits consumers’ purchase intention for unhealthy food, resulting in a negative food brand evaluation by consumers? Combined with the negative psychological effects of the PACE label on consumers in previous studies, such as negative emotions [38] and anticipatory guilt [46], a pioneering exploration of the negative effect of the PACE label on food brand evaluation was carried out in this study. Through analysis, it was found that the PACE label not only reduces consumers’ purchase intention for unhealthy food but also consumers’ food brand evaluation, which may lead brand owners to provide food-related PACE labels without motivation or passively, thus hindering the application of PACE labels in practice. The negative effect of the PACE label on consumers’ food brand evaluation is an important research direction that lacked attention in previous research. Therefore, this study fills the research gap regarding the PACE label and food brand evaluation to a certain extent.

This study also conducted analysis on the psychological mechanism behind the negative effect of the PACE label and a serial mediator model. The psychological mechanisms behind food labeling are rarely involved in research on food labeling [77]. Therefore, this study, after absorbing the regulatory focus theories [24] of psychology and consumer behavior, combining prevention focus [48] and anticipated enjoyment [25], provides an in-depth insight into this process. The process is as follows: Consumers have stronger prevention focus when stimulated by PACE labels, which can amplify the risks of food and negative information about it [32,48], thereby reducing their anticipated enjoyment of food brands; at the same time, because the anticipated enjoyment is an important factor for consumers to focus on food experience [53], consumers give a lower food brand evaluation. This study combines consumers’ psychological factors, such as regulatory focus theories and anticipated enjoyment, to deeply analyze the negative effects of PACE labels for the first time, which provides a new perspective for consumers’ psychology under the influence of PACE labels.

Thirdly, this study not only discusses the negative effects PACE labels produced on consumers with a prevention focus but also proposes moderating boundaries for the negative effects of PACE labels through reverse self-regulatory manipulation from the perspective of promotion focus [29] based on the bidirectionality of the regulatory focus [34]. Previous studies on the promotion focus mainly explore the behavioral characteristics of individuals drawing on advantages and averting hazards [27,28], such as the behavioral pattern of pursuing the maximum benefits in information search [32] and processing [33]. In this study, the promotion focus is used as an intervention tool to eliminate the negative effects of consumers after food label stimulation. The conclusions of this study have great inspiration and reference value for food brand owners and also propose as a new idea using contextual factors or cues [35,36] to improve consumers’ food decision-making behavior, so as to achieve the dual goals of lowering calorie intake and maintaining enterprise interests. It is also a research direction for future study to further consider the effect of food labels combined with psychological factors.

### 4.1. Practical Implication

The results of this study also have some practical implications for the government, health organizations, and food enterprises. Relevant studies have shown that only when their commercial interests are satisfied in business practice can enterprises promote the improvement of consumers’ health and welfare [23]. According to previous calorie labels, providing consumers with calorie labels for food can reduce consumers’ intake of high-calorie foods [8], which is conducive to improving consumers’ health and welfare and reducing consumers’ obesity-related diseases; however, it has an impact on the sales of non-healthy foods of the enterprise. If PACE labels have a negative impact on their food brand evaluation, enterprises have less motivation to promote the practical application of PACE labels.

Therefore, this study suggests that food enterprises can design cues for a promotion focus on shelves or food packaging to avoid the negative effects of PACE labels and improve food brand evaluation [60] such as posters about the pursuit of the inner “ideal self” [58] and situational cases where attention is paid to product advantages and benefits [61], thereby stimulating consumers’ tendency to promote directional decision-making and avoiding their negative brand evaluation. This study not only satisfies the government and health organizations’ pursuit of consumers’ health and welfare but also reduces the negative effect on enterprise brands, thus achieving the dual goals of “reducing calorie intake and maintaining enterprise interests”, and coordinating the dual demands of government and enterprise interests in the long term.

### 4.2. Limitations and Future Research Directions

The limitations of this study are summarized as follows. Firstly, this study was conducted through an online platform; however, consumption behaviors would be influenced by many other factors. To measure the effects of PACE labels on food consumption, field experiments can be more precise in revealing the relationship between PACE labeling strategy and changes in food consumption. Furthermore, preventing overweight is not just about changing eating habits; regular physical activity also can help manage weight. PACE labels show sports symbols and whether this can encourage consumers to exercise is open to debate. In other words, the effect of PACE labeling on solving the obesity problem can be addressed more deeply in other aspects.

Secondly, although studies have confirmed the effects for PACE labels on calorie intake, this study further found consumers may give a negative evaluation of a food brand when PACE labels are displayed on food products. On the other hand, we should notice that PACE labeling is still in its infancy. This strategy is rarely used in real markets. Governments have realized that improving consumers’ understanding of labels is crucial in helping them make better informed food choices [78]. Consumer purchase intention and food brand evaluation might have changed if PACE labels could be widely adopted in real markets. This could be studied in future research.

## 5. Conclusions

Through two experiments, which involved potato chips and chocolate products with standard calorie labels and PACE labels, this study confirmed that, compared with calorie labels, PACE labels not only reduce consumers’ willingness to purchase unhealthy foods but also have a stronger negative effect on food brand evaluation. Results also showed that consumers stimulated by PACE labels have a stronger prevention focus, weakening their anticipated enjoyment of food brands, and thus leading to lower food brand evaluation. In addition, when consumers have a promotion focus before choosing food, they cannot reduce their anticipated enjoyment and brand evaluation of the food with PACE labels. Therefore, the government and enterprises should not only pay attention to the inhibitory effect of PACE labels on customers’ purchase intention for unhealthy food but also focus on their negative impact on brand evaluation, so as to avoid the double negative effects on consumers regarding food purchase and brand evaluation.

## Figures and Tables

**Table 1:** General idea of experimental design.

Study	Hypothesis	Grouping	Major Variables	Stimulant	Control Variables
1	H1	Intergroups: label (PACE vs. calories)	Purchase intention, brand evaluation, regulatory focus, anticipated enjoyment	chickpea	Demographic
2	H2	Intergroup: 2 labels (PACE vs. calories) × 2 focus (promotion vs. control)		chocolate	Involvement in food choices

**Table 2:** Socio-demographics of consumers in Study 1–2.

Socio-Demographic Indicators		Study 1	Study 2
Variable	Definitions	Percentage	Percentage
Gender	Male	45.1%	36.9%
	Female	54.9%	63.1%
Age	≤20 years old	11.1%	8.9%
	21–30 years old	71.1%	52.5%
	31–40 years old	13.2%	28.3%
	≥41 years old	4.6%	10.3%
Education	Senior high school and below	2.0%	5.8%
	junior college	7.5%	16.7%
	bachelor’s degree	67.8%	65.6%
	post-graduate degree and above	22.7%	11.9%
Disposable income	2000 yuan and below	31.9%	18.6%
	2001–4000 yuan	24.7%	26.4%
	4001–6000 yuan	15.1%	21.7%
	6001 yuan and above	28.3%	33.3%
Valid sample size		304	360

**Table 3:** Main results of Study 1.

Variable	Definitions	PACE Group	Calories Group
Purchase intention	Mean (standard deviation)	3.66(1.455)	4.51(1.327)
	F	27.988 ***	
	partial η^2^	0.085	
	1-β	1.000	
Food brand evaluation	Mean (standard deviation)	4.01(1.400)	4.61(1.189)
	F	27.327 ***	
	partial η2	0.051	
	1-β	0.980	
Prevention focus	Mean (standard deviation)	5.13(0.947)	4.43(1.250)
	F	29.787 ***	
	partial η2	0.090	
	1-β	1.000	
Anticipated enjoyment	Mean (standard deviation)	4.18(1.458)	5.10(1.115)
	F	38.650 ***	
	partial η2	0.113	
	1-β	1.000	

Note: *** indicates significance at the 1% level.

**Table 4:** Main results of Study 2.

Variable	Definitions	Control		Promotion	
		PACE Group	Calories Group	PACE Group	Calories Group
Manipulation check	Mean (standard deviation)	5.18(1.196)		5.98(0.620)	
	partial η2	0.151			
	1-β	1.000			
Purchase intention	Mean (standard deviation)	4.40(1.490)	5.26(1.230)	4.58(1.388)	5.36(1.281)
	F	18.098 ***		14.987 ***	
Food brand evaluation	Mean (standard deviation)	4.55(1.241)	5.21(0.945)	5.15(1.131)	5.36(1.017)
	F	16.313 ***		1.692	
Prevention focus	Mean (standard deviation)	5.14(1.108)	4.11(1.601)	4.12(1.637)	4.10(1.580)
	F	25.271 ***		0.009	
Anticipated enjoyment	Mean (standard deviation)	4.84(1.526)	5.63(1.063)	5.48(1.103)	5.75(0.939)
	F	16.610 ***		3.256	

Note: *** indicates significance at the 1% level.

## Data Availability

All data included in this study are available upon request by contact with the corresponding author.
